# Supplementary material for: Vitamin D Modified DSS-Induced Colitis in Mice via STING Signaling Pathway
Source: Biology (Basel). 2025 Jun 18;14(6):715. doi: 10.3390/biology14060715 (PMC12190092; doi:10.3390/biology14060715)
Supplement: Supplementary file 1 [file biology-14-00715-s001.zip › Supplementary Materials S1: 16s-report of gut microbiota/Groups/heatmap/Group/non_norm_heatmap_log2.pdf]

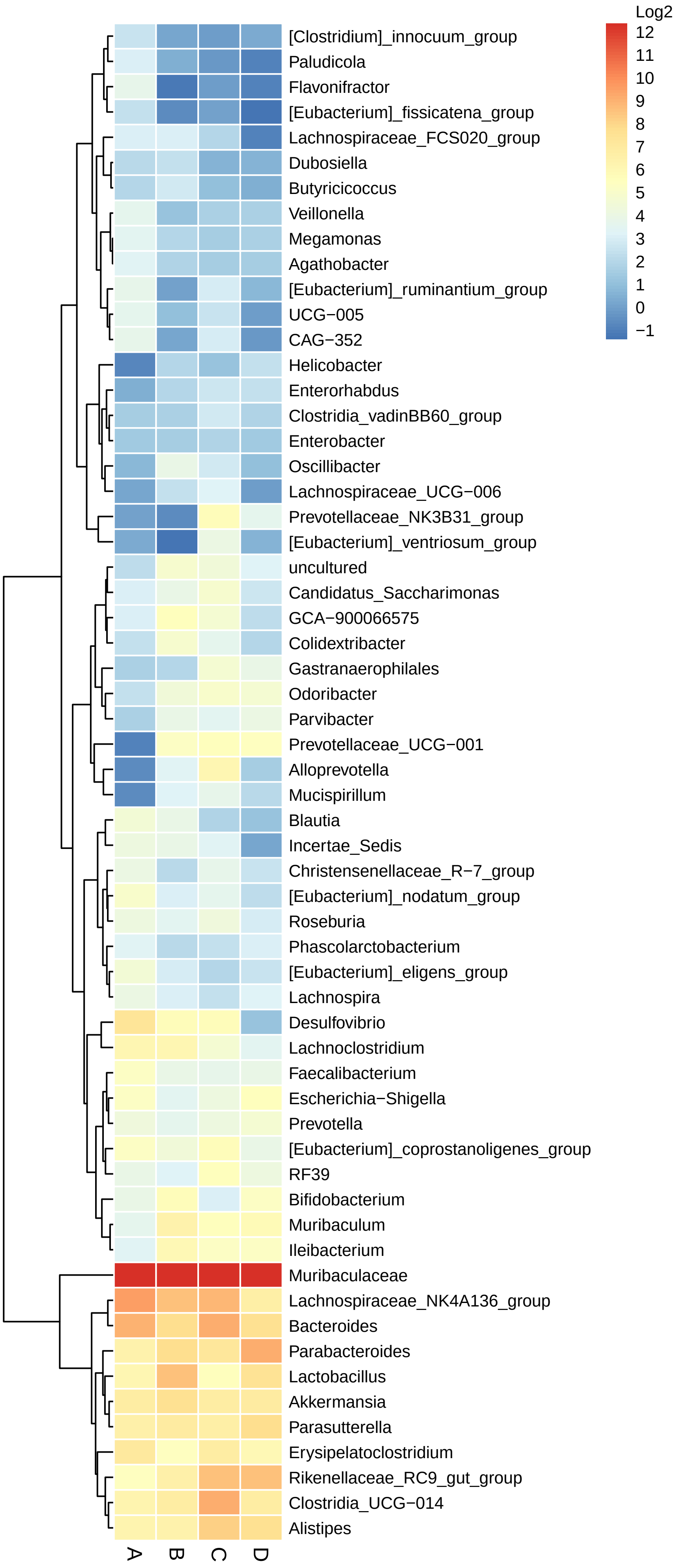
Log2
12
11
10
9
8
7
6
5
4
3
2
1
0
−1
[Clostridium]_innocuum_group
Paludicola
Flavonifractor
[Eubacterium]_fissicatena_group
Lachnospiraceae_FCS020_group
Dubosiella
Butyricicoccus
Veillonella
Megamonas
Agathobacter
[Eubacterium]_ruminantium_group
UCG−005
CAG−352
Helicobacter
Enterorhabdus
Clostridia_vadinBB60_group
Enterobacter
Oscillibacter
Lachnospiraceae_UCG−006
Prevotellaceae_NK3B31_group
[Eubacterium]_ventriosum_group
uncultured
Candidatus_Saccharimonas
GCA−900066575
Colidextribacter
Gastranaerophilales
Odoribacter
Parvibacter
Prevotellaceae_UCG−001
Alloprevotella
Mucispirillum
Blautia
Incertae_Sedis
Christensenellaceae_R−7_group
[Eubacterium]_nodatum_group
Roseburia
Phascolarctobacterium
[Eubacterium]_eligens_group
Lachnospira
Desulfovibrio
Lachnoclostridium
Faecalibacterium
Escherichia−Shigella
Prevotella
[Eubacterium]_coprostanoligenes_group
RF39
Bifidobacterium
Muribaculum
Ileibacterium
Muribaculaceae
Lachnospiraceae_NK4A136_group
Bacteroides
Parabacteroides
Lactobacillus
Akkermansia
Parasutterella
Erysipelatoclostridium
Rikenellaceae_RC9_gut_group
Clostridia_UCG−014
Alistipes
A
B
C
D
